# Comparative Review of the Responses of *Listeria monocytogenes* and *Escherichia coli* to Low pH Stress

**DOI:** 10.3390/genes11111330

**Published:** 2020-11-11

**Authors:** Talia Arcari, Marie-Lucie Feger, Duarte N. Guerreiro, Jialun Wu, Conor P. O’Byrne

**Affiliations:** Bacterial Stress Response Group, Microbiology, School of Natural Sciences, National University of Ireland Galway, H91 TK33 Galway, Ireland; TALIA.ARCARI@nuigalway.ie (T.A.); marielucie.feger@sfr.fr (M.-L.F.); duarte.guerreiro@nuigalway.ie (D.N.G.); J.WU4@nuigalway.ie (J.W.)

**Keywords:** *Listeria monocytogenes*, *Escherichia coli*, pH homeostasis, acid stress, acid sensing, organic acids, RpoS, Sigma B, decarboxylase, DNA damage

## Abstract

Acidity is one of the principal physicochemical factors that influence the behavior of microorganisms in any environment, and their response to it often determines their ability to grow and survive. Preventing the growth and survival of pathogenic bacteria or, conversely, promoting the growth of bacteria that are useful (in biotechnology and food production, for example), might be improved considerably by a deeper understanding of the protective responses that these microorganisms deploy in the face of acid stress. In this review, we survey the molecular mechanisms used by two unrelated bacterial species in their response to low pH stress. We chose to focus on two well-studied bacteria, *Escherichia coli* (phylum Proteobacteria) and *Listeria monocytogenes* (phylum Firmicutes), that have both evolved to be able to survive in the mammalian gastrointestinal tract. We review the mechanisms that these species use to maintain a functional intracellular pH as well as the protective mechanisms that they deploy to prevent acid damage to macromolecules in the cells. We discuss the mechanisms used to sense acid in the environment and the regulatory processes that are activated when acid is encountered. We also highlight the specific challenges presented by organic acids. Common themes emerge from this comparison as well as unique strategies that each species uses to cope with acid stress. We highlight some of the important research questions that still need to be addressed in this fascinating field.

## 1. Introduction

High proton concentrations, which define acidic environments, present a particular challenge for unicellular organisms since the protonation of biological molecules can affect their charge, structure, and function, which have potentially damaging consequences for the cell. Therefore, bacterial cells usually have homeostatic and protective mechanisms in place to counteract the inhibitory effects of low pH. Understanding how microbes sense and respond to acid stress is an important goal if we are to successfully control them, prevent infections, and to fully exploit them for biotechnological applications [1]. The goal of this review was to survey the molecular mechanisms that two unrelated well-studied bacteria use to combat the challenges presented by acidic pH.

We focus on *Escherichia coli*, perhaps the best studied of all bacterial species, which is a Gram-negative rod that belongs to the Gammaproteobacteria, and which typically occurs in nature as a commensal of the gastrointestinal (GI) tracts of mammals, birds, and reptiles. It is a highly diverse species that includes strains capable of causing infections in the GI tract and urinary tract, the latter being frequently associated with medical devices. Recent studies indicate that *E. coli* can also inhabit soil environments [2,3,4,5]. We also review the acid tolerance mechanisms of *Listeria monocytogenes*, a Gram-positive rod that belongs to the division Firmicutes, which is very well studied for its role as an intracellular food-borne pathogen of humans and cattle. In nature, *L. monocytogenes* is a saprophyte and is characterized by its tolerance to stresses such as low pH, elevated osmolarity, and bile salts and its ability to grow at refrigeration temperatures [6,7,8]. While these bacteria are phylogenetically unrelated, they share the ability to enter the host via contaminated food or water, and therefore both can encounter the gastrointestinal environment, including the extreme acid conditions prevailing in the stomach, as part of their life cycles. Several excellent reviews on the topic of acid tolerance have focused on one species or a number of closely related species [9,10,11]. Here we sought to compare and contrast the mechanisms used by these unrelated bacteria with the goal of highlighting common themes as well as individual strategies.

## 2. Maintaining Intracellular pH under Acidic Conditions

Under acidic conditions, bacteria use a variety of metabolic and homeostatic mechanisms to help maintain the cytoplasmic pH (pH_i_) within a range that is consistent with growth and survival. Both *Listeria monocytogenes* and *Escherichia coli* rely on several decarboxylation systems to protect the cell from a precipitous drop in pH. These systems depend on the activity of cytoplasmic pyridoxal-5’-phosphate (PLP)-containing amino acid decarboxylases which consume one proton and release one CO_2_ for every molecule of substrate amino acid, thus helping maintain the cytoplasmic pH [12]. With some exceptions, the reaction products are usually extruded from the cell through specific inner membrane antiporters in exchange for an extracellular amino acid. These decarboxylation systems include the glutamate decarboxylase (GAD) and the arginine decarboxylase (ADI) systems, which are present in both bacteria [13,14,15,16,17]. *E. coli* also has two additional amino acid decarboxylation systems: the lysine decarboxylase CadA [18] and the ornithine decarboxylase SpeF [19]. *L. monocytogenes* can catalyze two consecutive decarboxylation reactions to produce acetoin from pyruvate, consuming two protons in the process [20].

**Figure 1:**
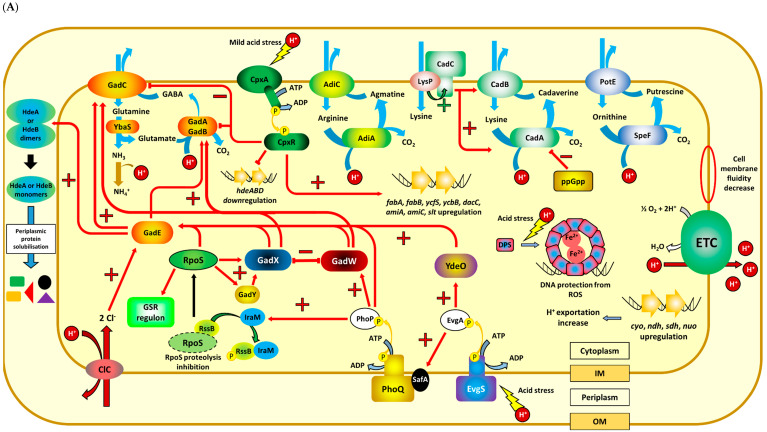

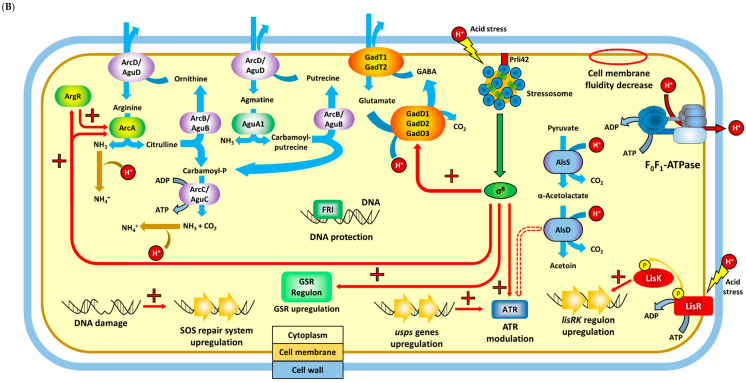
Schematic representation of the sensory, protective, and regulatory mechanisms triggered by environmental low pH conditions in *E. coli* (**A**) and *L. monocytogenes* (**B**). Red arrows represent upregulated (+) or downregulated (−) gene expression modulated by either periplasmic or cytoplasmic acidic pH. Decarboxylation and deamination reactions (represented by blue arrows) consume a proton (H^+^) and produce CO_2_, or produce ammonia (NH_3_), respectively. Ammonia can accept protons and yield ammonium (NH_4_^+^), thus contributing to pH homeostasis. (**A**) EvgS is thought to act as the periplasmic acidic pH sensor in *E. coli* and is responsible for the initiation of a complex signal transduction pathway that activates GadE and ultimately results in the upregulation of acid tolerance mechanisms. (**B**) It is hypothesized that the stressosome acts as the cytoplasmic pH sensor in *L. monocytogenes* and is responsible for the initiation of a signal transduction pathway that results in the release of σ^B^ and upregulation of the general stress response regulon (GSR).

In *E. coli*, the GAD system is composed of two isoenzymes, namely GadA and GadB, and the glutamate/γ-aminobutyric acid (GABA) antiporter GadC [14] (Figure 1A). The *gad* genes are induced in the stationary phase when cells are grown in rich media or during both growth phases when cells are grown in minimal media at pH 5.5 [21]. Decarboxylases have optimal enzymatic activities at acidic pH and their activities decrease sharply as pH increases. For GadA/B optimal pH is 3.7–3.8, indicating that these enzymes will be fully active under extreme acidic conditions [10]. Glutamine can also be imported into the cytoplasm by GadC and converted to glutamate by the amidohydrolase YbaS, with the release of an ammonia group. Free ammonia can also neutralize protons, thus Gln transport and YbaS also contribute to acid resistance in *E. coli* through the GAD system [22]. In *L. monocytogenes*, the GAD system comprises three homologous glutamate decarboxylases, GadD1, GadD2, and GadD3 and two antiporters GadT1 and GadT2, encoded at three distinct genetic *loci* [15,23] (Figure 1B). The *gadD1/T1* operon is part of the stress survival islet 1 (SSI-1), a hypervariable region of the *L. monocytogenes* genome that is absent in most serotype 4 *L. monocytogenes* clinical strains [24]. The GadD1/GadT1 system seems to be active during mildly acid stress whereas GadD2/GadT2 plays a role in survival under severe acid pH [25,26]. The *gadD3* gene is upregulated during acid stress and is σ^B^-dependent [23,27,28]. Moreover, the GadD3 system produces cytoplasmic GABA using the intracellular pool of glutamate, even in the absence of a functional GABA/Glu antiporter [23].

The arginine-dependent acid resistance system in *E. coli* consists of the acid-inducible arginine decarboxylase (AdiA) and the arginine/agmatine antiporter (AdiC) that exchanges extracellular arginine for the intracellular decarboxylation product agmatine [29,30,31,32] (Figure 1A). In *L. monocytogenes*, the ADI system consists of three enzymes and one membrane bound transporter: arginine deiminase (ArcA), catabolic ornithine carbamoyltransferase (ArcB, also known as AguB), carbamate kinase (ArcC, also known as AguC), and the arginine/ornithine antiporter ArcD (also known as AguD) [16] (Figure 1B). Arginine is converted into ornithine in a two-step reaction that also produces carbamoyl-phosphate and ammonia, which combines with intracellular protons to yield ammonium (NH_4_^+^), raising the cytoplasmic pH. Ornithine is transported out of the cell by the putative antiporter ArcD in exchange for a molecule of arginine. The *arcA* gene mediates the acid response in vitro and contributes to survival in human gastric fluid [33]. The carbamoyl-phosphate is further metabolized by ArcC into ammonium and carbon dioxide with the production of ATP. This ATP may contribute to the extrusion of cytoplasmic protons by the F_o_F_1_-ATPase, another mechanism that has been shown to play a role in the maintenance of pH homeostasis [34]. A lesser-known mechanism of acid stress resistance in *L. monocytogenes* is the agmatine deiminase (AgDI) pathway [17,33,35,36] (Figure 1B). The ADI and AgDI systems share the same genetic locus and some of these genes are probably involved in both pathways. ArcB was the first enzyme to be described as having both ornithine and putrescine carbamoyltransferase activities [17] (Figure 1B). Two putative agmatine deiminase homologues are present in the *L. monocytogenes* genome, namely *aguA1* and *aguA2*, but only AguA1 has been shown to have AgDI activity [35]. These enzymes catalyze the transformation of agmatine into putrescine, ammonium, carbon dioxide, and ATP, in a series of reactions that are analogous to those described for the ADI system. Putrescine is then exchanged with agmatine via the putative ArcD antiporter, but the activity and substrate specificity of this transporter remains to be determined.

Under mild acid stress (external pH ~5), CadA catalyzes the proton-consuming decarboxylation of lysine in *E. coli*, producing CO_2_ and the polyamine cadaverine, which is transported outside of the cell by the lysine/cadaverine antiporter CadB [18,37,38] (Figure 1A). The ornithine-dependent system, consisting of the ornithine decarboxylase SpeF and the ornithine/putrescine antiporter PotE, may also play a role under similar conditions, but its contribution to acid resistance in *E. coli* has not been unequivocally defined as for the other decarboxylases [19,39]. Another proton consuming process in *L. monocytogenes* is the production of acetoin from pyruvate. α-Acetolactate synthase (ALS) condenses two molecules of pyruvate to produce α-acetolactate, while consuming a proton. α-Acetolactate decarboxylase (ALD) decarboxylates acetolactate to form acetoin with the uptake of another proton [20] (Figure 1B). Expression of *alsS* and *alsD* is upregulated [40] and acetoin production rate is higher during acid stress [41]. This pathway also explains the role of thiamine in acid resistance in *L. monocytogenes*, since the enzymes involved in the conversion of pyruvate to acetoin both rely on this vitamin as a cofactor [42].

Mechanisms other than decarboxylation also play an important role in acid stress resistance. The F_o_F_1_-ATPase enzyme complex plays a major role in the regulation of intracellular pH in a number of bacteria [9], and the contribution of this proton translocating ATPase to acid resistance has also been studied in *E. coli* and *L. monocytogenes* [34,43,44]. A study by Cotter et al. [34] suggests that the F_o_F_1_-ATPase system plays a role in the acid tolerance response (ATR) of *L. monocytogenes.* Inhibition of the F_o_F_1_-ATPase by *N,N*’-dicyclohexylcarbodiimide (DCCD) prevents proton translocation out of the cell and severely affects the ability of bacteria to respond to acid stress. Additionally, some genes of the electron transport chain are upregulated in *E. coli* during acid stress [45]. These include cytochrome *bo* oxidase (*cyo* genes), NADH dehydrogenase II (*ndh* genes), succinate dehydrogenase (*sdh* genes), and NADH dehydrogenase I (*nuo* genes), suggesting an increased proton extrusion activity under acidic conditions [10] (Figure 1B). *E. coli* also uses two chloride transporters from the ClC family in the extreme acid resistance response [46]. Bacteria lacking these two genes have a severely compromised ability to withstand acidic conditions that resemble the gastric environment and the amino acid transport rates for the GAD and ADI systems are also affected [46]. Under low pH conditions, the ClC proteins function as a H^+^/Cl^−^ antiporter with a probable stoichiometric ratio of 2 Cl^−^/H^+^ [47] (Figure 1A). Chloride uptake contributes to the restoration of a negative-inside transmembrane potential following the reversal that occurs transiently during extreme acid stress when positive charged decarboxylation products accumulate in the cytoplasm [43]. In *E. coli* chloride ions also serve as positive allosteric effectors of the GadB glutamate decarboxylase [48]. The possibility that chloride ions might also influence decarboxylase activities in *L. monocytogenes* has not yet been investigated.

## 3. Protective and Repair Mechanisms against Acid Stress

Bacteria can deploy several protective and repair mechanisms to reduce the detrimental effects that low pH has on membranes, proteins and DNA. Many bacteria are capable of changing their membrane fatty acid profile in response to acidic conditions [49,50,51]. *E. coli* can increase the concentration of cyclopropane fatty acids (CFA) present in the cell membrane in order to decrease membrane permeability [52,53]. This requires a specific enzyme, the cyclopropane fatty acyl phospholipid synthase (CFAS), a soluble enzyme that is capable of transferring a methyl group from S-adenosyl methionine to unsaturated fatty acid molecules already present in the bacterial inner membrane [54,55]. The ability of *E.coli* to survive at pH 3.0 is correlated with the level of CFAs in the membrane [49] and *cfa*^-^ mutants are more sensitive to acid shock [52]. CFAs contribute to acid tolerance by decreasing membrane proton permeability and enhancing the ability to extrude protons [53]. How CFAs affect membrane properties like fluidity and permeability is not well understood. Recently, a molecular dynamics simulation suggested a dual role for CFAs: these lipids can stabilise cell membranes against adverse conditions and at the same time promote membrane fluidity [56]. The role that other cell membrane components play in acid stress resistance mechanisms in *E. coli* has been reviewed recently by Li et al. [57]. Other mechanisms to adjust membrane fatty acid composition under acid stress conditions have been described in Gram-positive bacteria. *L. monocytogenes* has an atypically high content of branched-chain fatty acids (BCFAs), and its ability to modulate the relative proportions of different BCFAs, allows the bacteria to adapt to moderate pH stress [58]. *L. monocytogenes* cells grown in the presence of various acids incorporated more saturated fatty acids and less BCFAs into their membranes, thus decreasing the membrane fluidity in response to acid stress [59]. Most recently the two component system CpxRA of *E. coli* has been shown to contribute to growth in moderate acidic conditions (pH 4.0–5.0) by upregulating the transcription of *fabA* and *fabB*, genes involved in the production of unsaturated fatty acids [60] (Figure 1A). Although the mechanisms underlying the modification of the membrane lipid composition in *E. coli* and *L. monocytogenes* under acidic conditions are somewhat different, the overall effect observed as a result of this change seem to be the remodeling of the membrane, maintaining its integrity and fluidity while conferring protection against acid stress.

Proteins in the bacterial periplasmic space are more vulnerable to acidic conditions than proteins in the cytoplasm, because of the relative permeability of the outer membrane porins to small molecules [61]. HdeA and HdeB are two periplasmic chaperones that play an essential role in *E. coli* during acid stress [62,63]. These proteins prevent protein aggregation induced by low pH and high periplasmic chloride concentrations and assist in the re-folding process of their substrates during pH neutralization [62,63,64,65]. Proteins in the periplasm cannot access the intracellular ATP pool and need to rely on an energy independent mechanism to fulfil their function. Both chaperones exist as functionally inactive dimers at neutral pH, but a decrease in pH triggers a dimer to monomer transition and partial protein unfolding, exposing a large hydrophobic surface that interacts with unfolded substrate proteins [62,63,66,67]. While HdeA is active at pH below 3, HdeB exhibits its highest chaperone activity at pH 4 [68]. The presence of both chaperones appears to enable *E. coli* to rapidly respond to a broader range of acid stress conditions, minimizing the irreversible aggregation of acid-unfolded proteins [68]. The cytoplasmic molecular chaperone Hsp31 also contributes to acid resistance in stationary phase *E. coli* [69]. Similarly, the chaperonin DnaK has been associated with acid tolerance in *L. monocytogenes*. A mutant strain lacking a functional *dnaK* gene exhibits reduced survival at low pH and high temperatures [70].

Dps is a DNA-binding protein expressed predominantly in starved *E. coli* cells and is highly conserved and broadly distributed throughout the bacterial domain of life [71]. In *E. coli*, Dps is one of the most abundant proteins during stationary growth and it protects cells against an array of stresses, including oxidative stress, starvation, heat shock and extreme pHs [72,73,74]. Dps expression is independently regulated by OxyR in exponentially growing cells and by the alternative sigma factor, sigma S (RpoS or σ^s^) and the histone-like integration host factor (IHF) during stationary phase [75]. Dps assembles into a dodecameric cage-like structure [76] and its protective effects are a consequence of three distinct biochemical mechanisms: Dps can bind and shield DNA from chemical damage, sequestrate Fe^2+^ ions in its core and oxidize iron by means of its ‘ferritin-like’ ferroxidase activity [74,77,78,79]. Under acid stress conditions, *dps^-^* mutants show 100-fold-greater sensitivity after 30 min at pH 2 [73] and DNA damage resulting from acid stress is greater in *dps* and *recA* mutants, highlighting also the importance of DNA repair in acid tolerance mechanisms [74]. In *L. monocytogenes*, the Dps homologue, Fri, is a major cold shock protein [80] that contributes to virulence and plays a role against multiple stresses [81,82,83,84] and whose expression is induced by low iron growth conditions [85]. Growth rate of a *fri ^-^* mutant strain is slightly reduced under hyperosmotic stress but is severely affected under acidic conditions (pH 5, HCl) [84]. These observations are similar to the results obtained in *E. coli*, suggesting an analogous role for both ferritin-like proteins in acid stress resistance in these bacteria.

Bacteria are able to respond to DNA-damaging agents by activating the SOS response, an inducible system that is involved in DNA repair. Two proteins play relevant regulatory roles in the SOS response: LexA, a repressor of the SOS regulon and RecA, a multifunctional protein involved in DNA recombination and repair that mediates auto-cleavage of LexA and induction of the SOS regulon [86,87]. In *E. coli*, acid stress produces DNA damage and *recA* mutants have a highly acid-sensitive phenotype [74]. The intracellular signal for the activation of this pathway includes the generation of single stranded DNA (ssDNA). However, not all the mechanisms that lead to the formation of ssDNA, and thus to SOS response induction, are well understood. Under acidic conditions, the mechanism resulting in the expression of SOS genes in *E. coli* might be explained by an alteration of the structure of LexA that leads to the formation of aggregates, degradation and de-repression of the SOS regulon genes [88]. In *L. monocytogenes*, a *recA^-^* mutant was also more sensitive to acid stress than the wild type strain [89], supporting the hypothesis that the SOS response plays an important role in the resistance of *L. monocytogenes* to acidic conditions.

## 4. Sensing and Regulatory Processes during Acid Stress

Although *E. coli* and *L. monocytogenes* both possess mechanisms that protect against low environmental pH, most of these mechanisms are deployed only when acidic conditions are encountered. In both bacterial species alternative sigma factors, RpoS (σ^s^) and SigB (σ^B^), respectively, contribute to acid resistance by reprogramming the transcriptional landscape during acidic conditions [90,91]. RpoS is responsible for the regulation of approximately 23% of the genes in the *E. coli* genome [92] and is regulated by a complex signal transduction pathway, initiated by the low pH sensor EvgS, which is the histidine kinase of the EvgS/EvgA two component system (Figure 1A). The sensing mechanism of EvgS is currently unknown but it has been shown that activation of this protein can be triggered by low pH and alkali metals [93,94,95]. A model for EvgS activation that involves structural changes in the EvgS dimer at low pH was proposed [96] and the His226 residue in the periplasmic domain has recently been shown to play a key role in the sensing mechanism [95]. A complex signal transduction cascade that involves SafA, the PhoQ–PhoP system, IraM, and the response regulator RssB leads to the inhibition of RpoS proteolysis [94,97,98,99] (Figure 1A). This pathway results in the upregulation of the expression of GadE, one of the main activators of the glutamate-dependent acid response system, responsible for the upregulation of the *gadA, gadBC*, *hdeA* and *hdeB* genes [98,100,101]. In addition to RpoS, a wide collection of regulators forms an intricate circuit that controls *gadE* expression under different growth conditions. These include the AraC-like family transcriptional regulators YdeO, GadX, GadW [98,102,103] and the global transcriptional regulator H-NS, that directly or indirectly regulates the expression of multiple key components of the acid resistance pathways [104,105].

In *L. monocytogenes*, σ^B^ is regulated by a signaling cascade composed by several proteins (RsbR, RsbS, RsbT, RsbU, RsbV, RsbW, and RsbX) (reviewed recently in [8,106]), and when activated it results in the upregulation of approximately 300 genes [107,108,109] that increase *L. monocytogenes* resistance towards lethal acid stress [23,25,26,110]. Several acid resistance related genes, including *argA*, *argR*, *gadD1*, and *gadD3* are regulated by σ^B^ [16,28,111,112] (Figure 1B). Although this signaling cascade is well studied, the molecular mechanism underpinning the acid sensing remains unknown. It is hypothesized that a supramolecular complex named as the stressosome (composed by RsbR, RsbS and RsbT) is responsible for the detection of environmental low pH, since this bacterium lacks the alternative pathway identified in *Bacillus subtilis* and *Bacillus cereus* that converges on RsbV [113,114,115]. Additionally, the stressosome has been found to be tethered to the cell membrane by Prli42, a small protein that has been shown to play a role in oxidative stress detection [116]; however, its role in acid sensing remains unstudied.

The activation of several acid resistance mechanisms such as the decarboxylation systems discussed above is triggered by a decrease in pH, both in the extracellular milieu and in the cytosol. In *E. coli*, each decarboxylase and their corresponding antiporters have different pH optima, although they are all active in the acidic pH range [100,117]; (reviewed in [10]). The oligomerization state of the decarboxylases is an important mechanism that contributes to the regulation of their activity. The pH-dependent conformational changes are responsible for the transition between their dimeric form and higher order oligomers (hexamers and decamers) [37,118,119]. AdiA activity is regulated by its oligomerization state, with a decamer being the main species at the optimal pH 5.2 and an increased concentration of inactive dimers at pH 7 [37,119,120]. A decrease in the intracellular pH (pH ~4) prompts a reversible structural rearrangement in GadB which disrupts the covalent bond between the His465 and PLP, resulting in the exposure of the active site and enabling the decarboxylation of glutamate while recruiting the protein to the membrane [118,121,122]. High-resolution X-ray crystal structures of GadC [123] and AdiC [117,124,125] have been solved and a model for substrate binding and conformational changes associated with the AdiC transport cycle has been proposed. Current knowledge on the structures, transport mechanisms, and regulation of these acid resistance associated antiporters have been recently reviewed [126]. Interestingly, the antiporters of each decarboxylation system in *E. coli* possess an invariant glutamic acid in the intermembrane domain that has been proposed to act as a pH sensor [117]. The model proposes that under extreme acidic conditions (between pH 2–3) AdiC assumes a conformation that exposes Glu208 to the acidic periplasm, resulting in its protonation. Once the conformation changes during substrate transport, Glu208 faces the less acidic cytosol returning to its deprotonated form [117]. Similarly, GadC is active at pH below 6.5 which promotes the rearrangement in the C-terminal region leaving the antiporter channel exposed [123]. This system has been previously reviewed [10,127].

The aforementioned invariant Glu is conserved in *L. monocytogenes* ArcD (Lmo0037 Glu205) and also in GadT1 and GadT2 (Lmo0448 Glu212 and Lmo2362 Glu213) (Arcari, Guerreiro and O’Byrne, unpublished data), which further supports the crucial role of this residue in low pH detection in antiporters from both bacterial species. In *E. coli* CadC, a ToxR-like DNA-binding transmembrane protein, has a periplasm-spanning C-terminal domain that acts as acid sensor through five negatively charged residues (Asp198, Asp200, Glu491, Glu468, and Asp 471) that are protonated under environmental low pH [128,129,130]. CadC activity is modulated by LysP, a lysine permease [131] that interacts with CadC enabling its activation in the presence of both lysine and low pH, resulting in the transcriptional activation of the *cadAB* operon [130,131,132,133,134]. CadA expression is induced at low pH, high lysine concentrations, and anaerobic conditions. Its enzymatic activity also depends on its oligomerization state and it is further regulated by the alarmone ppGpp [37,135]. The transcriptional regulator ArgR, a repressor of the arginine biosynthetic pathway in *L. monocytogenes*, is also involved in the acid stress response to lactic acid in this bacterium. The expression of σ^B^ was repressed by ArgR when bacteria were exposed to acidic conditions in an arginine-dependent manner [136] (Figure 1B). Finally, there is some evidence that the LisRK two-component system may also be involved in acid stress resistance in *L. monocytogenes* [137], but further studies will be required to better understand the role the LisRK regulon plays in acid stress resistance in this bacterium.

## 5. Short-Chain Organic Acid Stress

*E. coli* and *L.* *monocytogenes* are both food-borne pathogens, and their abilities to survive and thrive under organic acid stress are critical for the successful transmission from food to human beings. Food-grade organic acids are often used as food preservatives and organic acids are also fermentation products that can be naturally present in raw food or formed during fermentation processes. Moreover, upon ingestion of contaminated food products, the highly acidic stomach environment (pH 1–3) represents a great challenge to the survival of these bacteria. This is followed by a passage through mildly acidic to neutral environment in the intestine, where organic acids (e.g., bile acid) are abundant [138]. Weak organic acids are found to be more potent against bacteria especially under mildly acidic conditions when the acid groups are more likely to be protonated [139]. Depending on the type of acid, the modes of action can differ considerably; however, the common mechanism involves alteration of the pH_i_ and the accumulation of the weak acid anions in the cytoplasm. At low pH, the undissociated (protonated and uncharged) form of a weak acid diffuses through the cell membrane and dissociates intracellularly, acidifying the intracellular pH [139,140,141,142,143]. Meanwhile, dissociated acid anions accumulate within the cell and can cause turgor stress due to an increase in osmotic pressure [144]. Depending on the anion, accumulated acid might interrupt cellular processes [144,145], uncouple the proton motive force [146,147], decrease cell motility [148], and potentially disrupt the function of the membrane [149,150,151].

Both pathogenic (e.g., strain O157:H7) and non-pathogenic (e.g., strain K-12) *E. coli* upregulate genes involved in oxidative stress, cell envelope, cold shock stress, and iron and manganese uptake as a common response to HCl, acetate acid, and lactic acid stress [152]. Interestingly, strain-specific acid stress response genes mostly fall in the same functional categories as the universal acid response, indicating that different *E. coli* strains have evolved different genetic strategies to cope with the same stress [152]. In *L. monocytogenes*, a more significant overlap in gene regulation was observed when bacteria were exposed to these three acids, including upregulation of the GAD system, membrane modification, DNA damage repair, proteases and chaperonins, histidine synthesis, potassium uptake, and general stress proteins [153]. Both species upregulate cell envelope stress, oxidative stress, and DNA damage related genes, pinpointing the conserved nature of the acid stress response mechanisms among remotely related species.

In both *E. coli* and *L. monocytogenes*, lactic acid is a product of fermentative metabolism and it specifically induces the expression of a large set of genes, which overlaps significantly with the transcriptomic response to HCl [152,153,154,155], suggesting that similar stress response mechanisms are activated by these two acids. In *L. monocytogenes*, lactic acid uniquely induced differential expression of most genes among five tested weak acids (acetic acid, benzoic acid, citric acid, sorbic acid, and lactic acid) (Heavin and O’Byrne, unpublished data), and these findings are also supported by the observations from Tessema et al. [153]. In a recent transcriptomic study on *L. monocytogenes*, lactic acid exposure resulted in major change in gene expression (~2/3 of genome differentially expressed) [109]. Two lineage II strains (ST8 and ST121) shared most of the differentially regulated genes, which is probably not surprising due to the highly stable core genome of this species.

In contrast, acetate acid exposure results in differential regulation of a much smaller set of genes in both species compared to the expression profile observed upon exposure to other organic acids [152,153]. In *E. coli*, acetate specifically induces genes involved in metabolism [156,157], and multidrug and aromatic carboxylic acid efflux [152]. It is however surprising that genes in the acid fitness island (AFI), which includes *slp*, *hdeB*, *hdeA*, *gadE*, *mdtE*, and *gadA*, were down regulated in *E. coli* K-12 when exposed to acetic acid [152]. Acetate accumulation has been investigated in *E. coli* and concentrations can get as high as 230 mM inside the cell when the extracellular concentration is only 8 mM. This turgor stress is thought to be partially relieved by reducing intracellular glutamate concentration [144]. Further investigations showed that the inhibitory effect of acetic acid on *E. coli* in chemically defined media is largely attributed to interruption of methionine biosynthesis and concomitant accumulation of the toxic metabolite homocysteine [145], which is itself inhibitory because it perturbs branched chain amino acid biosynthesis [158]. Recently, the transhydrogenase UdhA was identified as an important source of NADH for the growth of *E. coli* on acetate and plays a positive role in the regulation of expression of the GAD system [159]. In *L. monocytogenes*, two studies have revealed significant strain to strain variation in the transcriptomic response to acetate [40,41]. Nevertheless, upregulation of branch-chain fatty acid synthesis related genes appears as one common response to acetate stress in this species [40].

Benzoic acid is a partial uncoupler that disrupts the proton motive force in *E. coli* [160]. Exposure to benzoic acid in *E. coli* induces the AFI genes and the GadE regulon. Interestingly, evolution in the presence of growth permissive concentration of benzoic acid selected for strains that lost GAD activity [160,161]. This phenomenon is thought to be explained by the fitness cost of activating protective mechanisms that fail to provide benefit. Indeed, the GAD system was found to provide protection against lethal acetate acid stress in *E. coli* [152] and does not contribute significantly to the growth of *L. monocytogenes* in the presence of acetate, benzoate, and sorbate [162]. In the latter species, benzoic acid specifically induces genes involved in carbohydrate metabolism, transport/binding proteins, lipoproteins, and multidrug efflux pump MdrL (Heavin and O’Byrne, unpublished data). Another important group of organic acids that are present in the human GI tract are bile acids. *E. coli* can reduce the toxicity of bile acids by means of a 7α -hydroxysteroid dehydrogenase [163,164] and their active efflux via the AcrAB and EmrAB multidrug efflux systems [165]. *L. monocytogenes* encodes a bile salt hydrolase which is absent in the non-pathogenic species *L. innocua* [166]. Similar to *E. coli*, multidrug efflux systems were also found to be involved in bile acids stress response in *L. monocytogenes* [167,168]. An increased sensitivity to bile acids was observed in *L. monocytogenes* at acidic pH [169] but bacteria displayed an enhanced resistance to bile acids when bacteria were pre-adapted to several stresses [170]. Numerous mechanisms of bile acids stress response in *L. monocytogenes* were reviewed by Davis et al. [171]. Interestingly, genes related to osmotic stress are found differentially regulated under organic acid stress in both species. In *E. coli*, genes involved in proline accumulation and osmotically inducible genes are upregulated in O157:H7 [152,156]. In *L. monocytogenes*, *sigL* and genes involved in carnitine/betaine and potassium uptake are upregulated [153]. SigL and the carnitine transporter are indeed confirmed to play a role in organic acid stress response [172]. In addition, multiple osmolyte transporter (-like) systems have been demonstrated to contribute to bile acids tolerance [173,174].

## 6. Conclusions and Future Perspectives

Bacteria have evolved sophisticated mechanisms to survive and grow under acidic conditions. The particular solutions used by a given species to the problems created by high proton concentrations clearly depends on its natural environment and on its unique physiology and metabolic traits. *E. coli* and *L. monocytogenes* are unrelated phylogenetically yet acidity presents common challenges to both; protecting the internal pH, protecting the genetic material, and critical enzyme function. The different mechanisms deployed by these two bacteria under acidic conditions are summarized in Table 1. They both have very finely tuned regulatory circuits to activate the expression of protective and homeostatic functions. Alternative sigma factors play a critical role in reprogramming the transcriptional landscape in both species, RpoS in *E. coli* and σ^B^ in *L. monocytogenes.* In both cases, a key research question remains to be answered; how is the initial acid-specific signal detected by the cell? Answering this question has proven difficult partly because the signal could be either direct (e.g., proton concentration) or indirect (e.g., protein unfolding, altered concentration of some ion or metabolite, etc.).

As we have seen, both species use amino acid decarboxylation as means of buffering the cytoplasmic pH against a potentially lethal drop in pH. The regulation of these systems in *E. coli* is very complex, which likely highlights the importance to fitness of getting this decision right. Given the diverse roles that glutamate plays in the cell this should probably not come as a surprise. In *L. monocytogenes* the presence of three glutamate decarboxylase systems highlights its importance to acid tolerance but leaves us with much to learn about how they are collectively or independently regulated. It is also clear that strain to strain differences exist within this species in the extent to which they rely on each of the three glutamate decarboxylases systems [24,175]. Given the critical role of these systems in acid tolerance, they represent a potentially useful target for the development of next-generation antimicrobials. Preventing their activity could potentially reduce the growth and survival of pathogenic strains of either *E. coli* or *L. monocytogenes* in acidic food products and furthermore could serve to reduce the likelihood of these pathogens surviving in the acidic conditions of the stomach after ingestion. Indeed, one recent study has shown that the GAD system of *L. monocytogenes* can be inhibited by maleic acid [176], suggesting that small molecule inhibitors might be a possibility.

Other common themes that emerge from this comparative review of the protective systems used by these bacteria include mechanisms to limit proton ingress, protection of proteins, and nucleic acids from damage. Both species have specific mechanisms to alter the composition of their cell envelope in response to acidic environments. The role of cyclopropane fatty acids in reducing the permeability of the membrane of *E. coli* to protons is clear. The changes that occur in the lipid bilayer of *L. monocytogenes* are known, but further research is needed to determine how these changes protect the cell and to understand the regulatory processes involved. Preserving the function of critical enzymes in the cell is likely to be the problem faced by all microbes exposed to extreme acid stress. The role of the extra-cytoplasmic protein chaperones HdeA and HdeB in *E. coli* in preserving the integrity of periplasmic proteins at low pH is now well established, but the role of intracellular chaperones and proteases is less clear at this stage. DNA protection and repair appear critical in both species, although the precise mechanisms of how acidic pH leads to DNA damage still needs further investigation. Perhaps the greatest difference regarding the acid stress responses found in these two bacteria lies within the sensing mechanisms used to detect the acidic environments. Whereas *L. monocytogenes* is capable of sensing acid stress through the stressosome, *E. coli* lacks this supramolecular complex and relies mainly on the EvgSA two-component system to detect low pH and activate the signal transduction pathways that leads to the upregulation of acid stress response genes.

Overall, this field still has important unanswered questions that, if addressed, have the potential to give major new insights into the biology of these bacteria. Although the problems are fundamental in nature, the answers might lead to innovative solutions to controlling these organisms, particularly in the food chain where their presence can cause significant economic and public health issues for mankind.

## Figures and Tables

**Table 1:** Summary of mechanisms deployed by *E. coli* and *L. monocytogenes* to cope with acid stress.

Mechanism/Response	*E. coli*	Key References	*L. monocytogenes*	Key References
**pH homeostasis**				
Proton consuming reactions	GAD system	[13,14,118,123]	GAD system	[15,23,25,26]
	ADI system	[31,32,119,124,125]	ADI system	[16,17,33]
			AgDI system	[17,35,36]
			Acetoin production	[20,41]
	CadA	[18,37,38,128,129]		
	SpeF	[19,39]		
Proton extrusion mechanism	ClC	[46,47]		
	ETC	[10,45]		
	F_o_F_1_-ATPase	[44]	F_o_F_1_-ATPase	[34]
**Protection and repair**				
Membrane composition	CFAs	[52,53,54,55]	BCFAs	[58,59]
Chaperones	HdeA, HdeB	[62,63,67,68]	DnaK	[70]
	Hsp31	[69]		
	Dps	[72,74,76]	Fri	[80,81,82,84]
DNA damage	SOS response	[86,87,88]	SOS response	[89]
**Sensing and regulatory**				
Two-component systems	EvgAS	[95,96,97,98,99]	LisRK	[137]
	PhoQ-PhoP	[99]		
	CpxRA	[60]		
Sensory hub			Stressosome	[8,105,115]
Alternative Sigma factors	RpoS	[91,92]	SigB	[8,106]
Regulators	GadE ‘circuit’	[100,101,102,103]	ArgR	[16,135]
**Response to short-chain organic acid**				
Lactic acid		[152]		[109,153,155]
Acetic acid		[144,145,152,156,157,158]		[40,41,153]
Benzoic acid		[160,161]		[162]
